# Deletion of hepatic growth hormone receptor (GHR) alters the mouse gut microbiota by affecting bile acid metabolism

**DOI:** 10.1080/19490976.2023.2221098

**Published:** 2023-06-12

**Authors:** Zichao Yu, Yu Wang, Fang Zhang, Rui Ma, Xiaoyu Yang, Kun Yang, Ai Mi, Liyuan Ran, Yingjie Wu

**Affiliations:** a Shandong Provincial Hospital Affiliated to Shandong First Medical University & Shandong Academy of Medical Sciences, School of Laboratory Animal & Shandong Laboratory Animal Center, Medical Science and Technology Innovation Center, Shandong First Medical University & Shandong Academy of Medical Sciences, Jinan, China; b Institute of Genome Engineered Animal Models for Human Diseases, Dalian Medical University, Dalian, China; c National Center of Genetically Engineered Animal Models for International Research, Dalian Medical University, Dalian, China

**Keywords:** Growth hormone, Growth hormone receptor, Tissue specific gene knockout, Bile acid, Gut microbiota, Microbial metabolites

## Abstract

Both growth hormone (GH) and gut microbiota play significant roles in diverse physiological processes, but the crosstalk between them is poorly understood. Despite the regulation of GH by gut microbiota, study on GH’s influence on gut microbiota is limited, especially on the impacts of tissue specific GH signaling and their feedback effects on the host. In this study, we profiled gut microbiota and metabolome in tissue-specific GHR knockout mice in the liver (LKO) and adipose tissue (AKO). We found that GHR disruption in the liver rather than adipose tissue affected gut microbiota. It changed the abundance of *Bacteroidota* and *Firmicutes* at phylum level as well as abundance of several genera, such as *Lactobacillus*, *Muribaculaceae*, and *Parasutterella*, without affecting α-diversity. Moreover, the impaired liver bile acid (BA) profile in LKO mice was strongly associated with the change of gut microbiota. The BA pools and 12-OH BAs/non-12-OH BAs ratio were increased in the LKO mice, which was due to the induction of CYP8B1 by hepatic *Ghr* knockout. Consequently, the impaired BA pool in cecal content interacted with gut bacteria, which in turn increased the production of bacteria derived acetic acid, propionic acid, and phenylacetic acid that were possible to participate in the impaired metabolic phenotype of the LKO mice. Collectively, our findings suggested that the liver GH signaling regulates BA metabolism by its direct regulation on CYP8B1, which is an important factor influencing gut microbiota. Our study is significant in exploring gut microbiota modification effects of tissue-specific GH signaling as well as its involvement in gut microbiota–host interaction.

## Introduction

The homeostatic relationship between host and gut microbiota is of significant importance for the host’s health, and the dysbiosis of gut microbiota is associated with many metabolic diseases, such as nonalcoholic fatty liver disease (NAFLD) and obesity.^1^ Previous studies have highlighted the roles of the liver-, muscle- and adipose tissue-gut microbiota axes in host metabolism.^2–4^ Notably, all of these tissues are targets of growth hormone (GH), which prompted us to explore whether there is a crosstalk between GH signaling and gut microbiota and the mediators involved in their interaction.

GH has profound effects on regulating growth, metabolism, immunity, homeostatic processes, and aging.^5–8^ It functions by directly binding to its membrane receptor (GHR) that activates several signaling cascades and exerts the pleiotropic effects synergistically with or independent of insulin-like growth factor 1 (IGF1).^7^ The impaired GH signaling is considered to cause many metabolic diseases. It has been identified that people with lower serum GH has higher prevalence of NAFLD and obesity,^9,10^ and Laron syndrome patients caused by *GHR* mutation also develop NAFLD.^11^ Although the association of GH signaling with metabolic diseases has been proposed previously, GH’s functional roles especially that involved in the host–gut microbiota interaction are still undefined. In spite of the GH/IGF1 regulation by gut microbiota in many animal models including mice, chicken, lamb, and *Drosophila*,^12–18^ the effects of GH signaling on gut microbiota were less studied. Emerging evidences has implied that fluctuation of GH level can influence the gut microbiota. Jensen *et al*. demonstrated that global GH excess and deficiency affected the gut microbiota in mature adult and middle-aged mice.^19,20^ Moreover, the gut microbiota in patients with GH-secreting pituitary adenoma is significantly altered and strongly associated with GH/IGF-1 axis.^21^ Therefore, exploring the roles of GH signaling in gut microbiota regulation can provide a better understanding of the pathophysiological process of diseases related to impaired GH axis. However, the mechanism by which GH signaling influences gut microbiota is still unclear.

Bile acids produced in the liver can affect gut microbial growth in both direct and indirect ways,^22,23^ thus mediating the interaction between the host and gut microbiota. Bile acids are synthesized via cytochrome P450 (CYP) enzyme-mediated oxidation of cholesterol. CYP7A1 and CYP8B1 are key enzymes in bile acid synthesis with the former as the rate-limiting enzyme and the latter as a crucial regulator that determines the ratio of 12-OH bile acids to non-12-OH bile acids.^24^ Accumulating studies have reported that the increased 12-OH bile acid ratio or upregulation of CYP8B1 leads to human diseases, such as NAFLD, obesity, and inflammatory bowel disease (IBD),^25–27^ which makes CYP8B1 a viable therapeutic target for metabolic diseases. GH signaling has been regarded to regulate the cholesterol and lipid metabolism. However, its role in bile acid metabolism is not clear. Therefore, investigating the mechanisms of bile acid metabolism regulation by GH signaling and its subsequent effects on gut microbiota may extend our understanding of pathophysiological roles of GH signaling and facilitate the exploration of therapeutic targets for metabolic diseases.

The liver and adipose tissue are import target of GH, but the GH effects mediated by GHR are tissue specific. For example, *Ghr* disruption in the adipose tissue resulted in fat mass increase, while it was resistant to high fat induced hepatic steatosis.^28^ However, the hepatic *Ghr* disruption led to the spontaneous fatty liver under regular chow.^29,30^ The tissue-specific *Ghr* knockout mice have become useful tools to better understand the direct actions of GH in individual tissue.^31^ However, the effect of tissue-specific GH signaling on gut microbiota is unknown. In this study, by employing the GHR knockout mice specifically in the adipose tissue and liver, we demonstrated the effects of tissue-specific GH signaling on gut microbiota and the role of liver GHR in regulating bile acid metabolism. Our data uncovered the mechanism of liver GHR in modulating gut microbiota by bile acid metabolism regulation as well as the potential feedback of the altered gut microbiota.

## Results

### Absence of liver *Ghr* affects the gut microbiota

**Figure 1.**
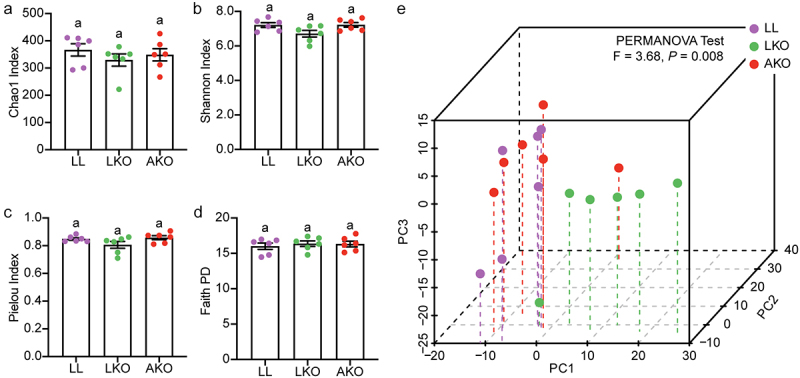
Analysis of the α-diversity (a-d) and beta-diversity (e) of gut bacterial community. Lowercase letters above the error bars indicate significant differences among groups.

To determine the effects of tissue-specific GH signaling on gut microbiota, the diversities of gut bacterial community from GHR^flox/flox^ (LL), AKO and LKO mice were compared. Neither the adipose tissue nor the liver *Ghr* knockout affected the α-diversity, including Chao1, Shannon, Pielou, and Faith PD indices, compared with the LL group (Figure 1a-d). For the β-diversity based on ASVs abundance, the samples in the LKO group were clustered together, while those in the LL and AKO groups formed the other cluster in the PCA plot (Figure 1e). PERMANOVA test result indicated that the bacterial community structure in LKO was significantly different from both the LL and AKO groups, but the difference between LL and AKO groups was minor and not significant, which was indicated by the lowest F value with a *P* value of 0.431 (Table S1).

**Figure 2.**
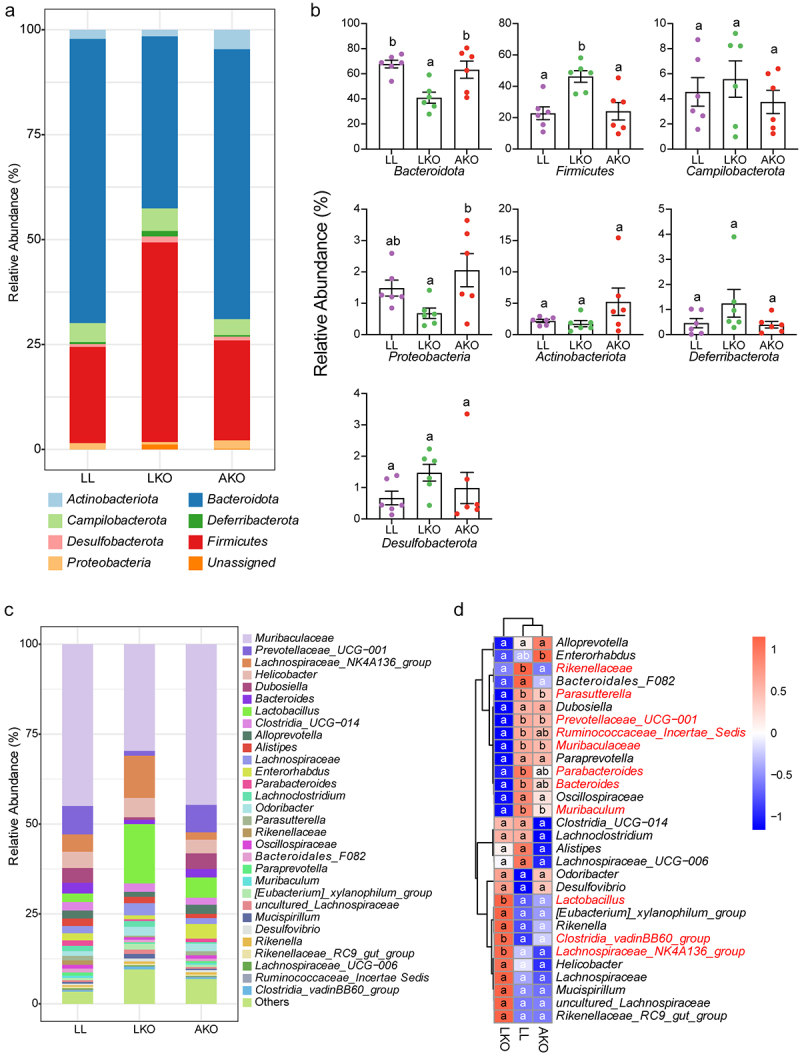
Taxonomic composition of gut bacterial community in LL, LKO, and AKO mice. (a) Composition of the bacterial community in different mouse groups at the phylum level. (b) Comparison of the relative abundance of these phyla among different mouse groups. (c) Composition of the top 30 most abundant genera in different mouse groups. (d) Heatmap illustrating the variations in top 30 genera among different mouse groups. The significantly altered genus were shown in red in the heatmap. Different lowercase letters above the error bars and in the heatmap indicate significant differences among groups.

The taxonomic composition variation in bacterial community was analyzed at the phylum and genus levels. Seven phyla were detected in the cecum, and all the bacterial communities were dominated by *Bacteroidota* and *Firmicutes*, followed by *Campilobacterota*, *Actinobacteriota*, and *Proteobacteria* (Figure 2a). There was no significant difference in the abundances of all the detected phyla between the LL and AKO groups (Figure 2b). However, the abundance of *Bacteroidota* was significantly decreased, and that of *Firmicutes* was significantly increased in the LKO group. In addition, the abundance of *Proteobacteria* was significantly lower in the LKO group than in the AKO group. The composition and proportion of the top 30 most abundant genera was shown in Figure 2c, and *Muribaculaceae* was the most abundant in all the groups. The genus composition in LL and AKO groups was similar, since they were in the same branch based on the cluster analysis in the heatmap (Figure 2d). In addition, compared with the LL group, the abundances of several genera were significantly altered in the LKO group. *Lactobacillus*, *Clostridia vadinBB6 group* and *Lachnospiraceae NK4A136 group* were significantly enriched, while the abundances of *Rikenellaceae*, *Parasutterella*, *Prevotellaceae UCG-001*, *Ruminococcaceae*, *Muribaculaceae*, *Parabacteroides*, *Bacteroides* and *Muribaculum* were significantly decreased in the LKO group. Similar with that at the phylum level, there was also no significant difference in these genera except for *Rikenellaceae* between the LL and AKO groups.

### Variation of gut microbiota is correlated with the impaired metabolism in hepatic GHR deletion mice

Deletion of GHR in the liver and adipose tissue both affected the metabolism. The LKO mice were characterized by heavier liver, and AKO mice were characterized by heavier brown and white adipose tissue, although there was no difference among their body weight (Figure S1A-C). Moreover, the level of serum TG, TCHO, LDLC, and HDLC as well as liver TG was significantly increased in the LKO mice, while there was no difference between the LL and AKO mice (Figure S1D-E). The H&E staining of WAT and liver was consistent with the above results, which showed that the AKO mice had expanded adipocytes and the LKO mice had significant increase in hepatic steatosis (Figure S1H-I). In addition, the glucose homeostasis was also changed by tissue-specific *Ghr* knockout. Compared with the LL mice, the insulin tolerance test (ITT) was significantly impaired in the LKO mice, while improved in the AKO mice (Figure S1F-G). The correlation between the metabolic phenotype and gut microbiota was analyzed. In the LKO mice, many genera, such as *Muribaculum*, *Parabacteroides*, and *Parasutterella* were significantly negatively correlated with most of the metabolic indices, especially with those related to lipid metabolism. *Lactobacillus* was positively correlated with the BAT weight, ITT, and the lipid metabolism related indices (Figure S1J). Moreover, all of these bacteria were significantly altered in the LKO mice. In the AKO mice, only a few genera were correlated with a small number of metabolic indices (Figure S2), but their abundance was not significantly different from that in the LL mice. Therefore, the results suggested that the change of gut microbiota in the LKO mice was correlated with its impaired metabolic phenotype.

### Hepatic bile acid profile is associated with gut microbiota shifts

**Figure 3.**
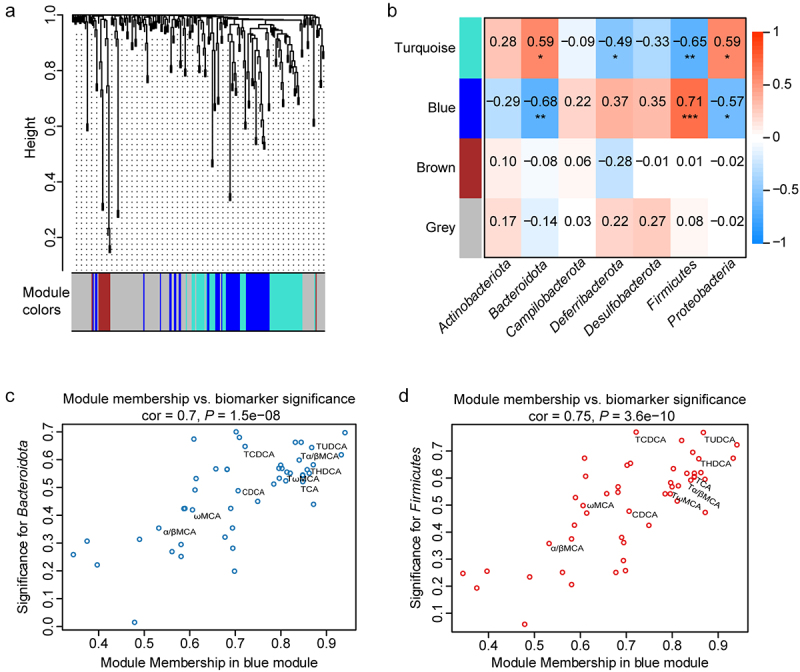
Correlation analysis of gut bacterial phyla and liver metabolites by WGCNA. (a) Metabolite modules obtained based on the 214 metabolites in the liver in LL, LKO, and AKO mice. (b) Heatmap presenting module–trait relationships based on the Pearson method. Each row corresponds to a module eigengene (ME) and each column to the abundance of a bacterial phylum. The corresponding correlation coefficient is displayed at the top of the cell, and corresponding *P* value for each module is displayed by the star. *: *P* < 0.05, **: *P* < 0.01, ***: *P* < 0.001. (c-d) The relationship between module membership (MM) in blue module and biomarker significance (BS) of Bacteroidota (c) and Firmicutes (d). The MM of a node represents the correlation between the node profile and the ME, and the BS of a node represents the correlation between the node profile and a given trait. The greater absolute value of the MM indicates the node is more highly representative of the module, and the greater absolute value of the BS represents the node is more biologically significant.

GH plays an important role in regulating the metabolism in adipose tissue and liver. Therefore, targeted metabolomics assay for adipose tissue and liver of all the mouse groups were performed to investigate the mechanisms involved in gut bacterial community shifts mediated by tissue-specific *Ghr* knockout. A total of 214 and 196 metabolites were detected in the liver and adipose tissue, respectively (Table S2 and Table S3). WGCNA was performed to identify metabolic modules clustering and explore the correlation of bacterial phyla and metabolites. For the liver metabolic profile, there were four modules obtained using the clustering algorithm (Figure 3a), and 54.1% of the metabolites were included in the color modules, while the others that did not displayed the obvious co-expression trend were included in the gray module. The associations between the modules and bacterial phyla indicated that the blue module had the most significant and strongest correlation with the phyla of *Bacteroidota* and *Firmicutes* (Figure 3b). In-module analysis (Figure 3c-d) also showed highly significant correlation between the module membership and biomarker significance of *Bacteroidota* and *Firmicutes*. In addition, 47.3% (9/19) of the total bile acids were clustered in the blue module, and most of them had high correlation with the changes of *Bacteroidota* and *Firmicutes*, which were indicated by their distribution in the top right quarter of the module membership vs. biomarker significance plots (Figure 3c-d). There were also four modules obtained by WGCNA from the metabolites in adipose tissue, and the brown module had the significant correlation with *Bacteroidota* and *Firmicutes* (Figure S3A-B). However, the correlations between the module membership and gene significance were not significant. Even so, most of the bile acids showed relatively high correlation with the changes of *Bacteroidota* and *Firmicutes* (Figure S3C-D). These results suggested that the hepatic bile acids were associated with the variation of gut bacterial community.

### Bile acid metabolism is disturbed by hepatic *Ghr* absence

**Figure 4.**
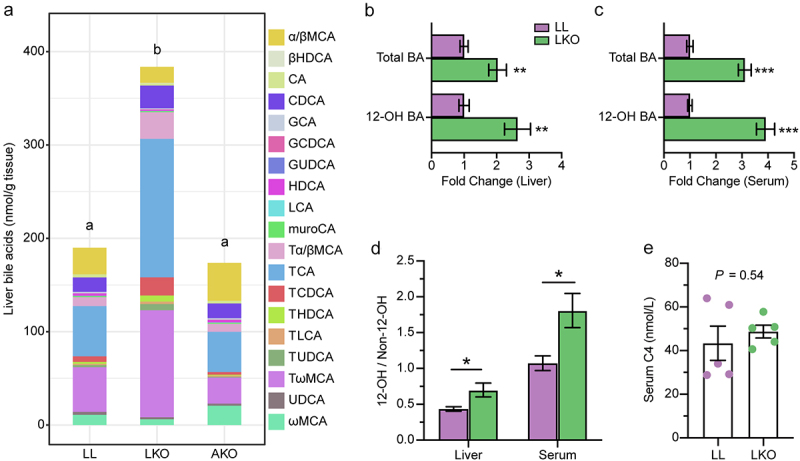
Change of the bile acid profile in different mice. (a) Composition of the liver bile acid pool in LL, LKO, and AKO mice. (b-c): Fold change of total bile acid and 12-OH bile acids in the liver (b) and serum (c) between the LL and LKO group. (d) Ratio of 12-OH bile acids to non-12-OH bile acids in the liver and serum of LL and LKO mice. (e) Content of serum C4 in LL and LKO mice. *: *P* < 0.05, **: *P* < 0.01, ***: *P* < 0.001.

Bile acid is synthesized by hepatocyte, hence we further analyzed the bile acid profile in the liver of the three mouse groups based on the targeted quantitative metabolomics data. Nineteen bile acids were detected in the liver, and the total bile acid content was significantly higher in the LKO group than in the LL and AKO group (Figure 4a). PCA plot and PERMANOVA test indicated that there was no significant difference in the bile acid profile between LL and AKO groups, whereas the bile acid profile in the LKO group was significantly different from the other two groups (Figure S4A-B). Consistent with the PCA analysis, the heatmap showed that many conjugated bile acids were significantly enriched in the LKO group rather than the AKO group (Figure S4C-D), while the unconjugated bile acids were not varied among these groups (Figure S4E). Furthermore, the 12-OH bile acids were significantly enriched in both the liver and serum of LKO mice. Although content of total bile acid was increased in the LKO mice, the fold change of 12-OH bile acids was higher (Figure 4b-c). Moreover, the ratio of 12-OH bile acids to non-12-OH bile acids was significantly higher in both the liver and serum of the LKO mice (Figure 4d) and the content of serum 7α-hydroxy-4-cholesten-3-one (C4) (a marker for CYP7A1 activity) was not changed (Figure 4e).

### Hepatic GHR regulates *Cyp8b1* expression

**Figure 5.**
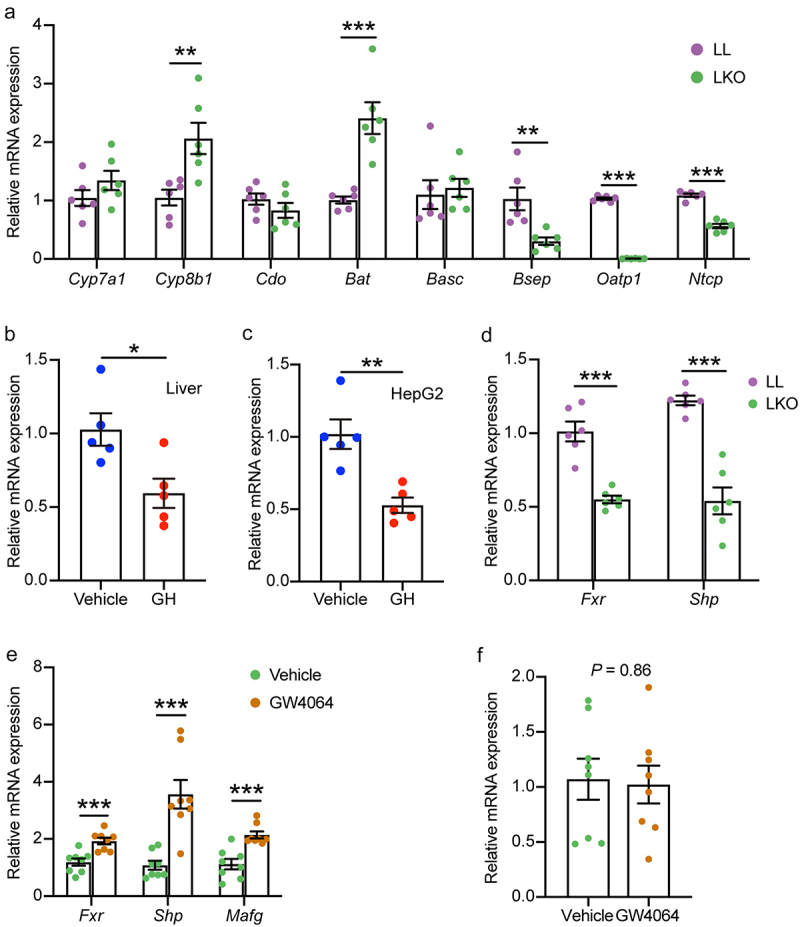
Analysis of bile acid metabolism regulation by hepatic GH signaling. (a) Expression level of genes related to bile acid metabolism in the liver of LL and LKO mice. (b-c) Expression level of *Cyp8b1* in rhGH treated HFD fed mice (b) and HepG2 cells (c). (d) Expression level of hepatic *Fxr* and *Shp* in LL and LKO mice. (e) Expression level of hepatic *Fxr* and its downstream *Shp* and *MafG* in LKO mice after GW4064 treatment. (f) Expression level of hepatic *Cyp8b1* in the GW4064 treated LKO mice. *: *P* < 0.05, **: *P* < 0.01, ***: *P* < 0.001.

The expression level of genes related to bile acid metabolism was examined to explore the bile acid metabolism regulation of hepatic GHR. Consistent with the increased 12-OH/non-12-OH ratio, the expression of *Cyp8b1* that determines the ratio of 12-OH bile acids to non-12-OH bile acids was significantly upregulated in the liver of LKO mice (Figure 5a). However, the expression level of *Cyp7a1* encoding the rate-limiting enzyme for BA synthesis was not changed, which was in line with the serum C4 content. These results suggested that the highly induced bile acids in the LKO mice attributed to the upregulation of *Cyp8b1*. In addition, the expression of taurine biosynthesis-related cysteine dioxygenase gene (*Cdo*) and bile acid conjugation-related bile acid:CoA synthase gene (*Bacs*) was not changed, but the bile acid-CoA:amino acid N-acyltransferase gene (*Bat*) that conjugates bile salts to taurine or glycine was significantly induced. The bile salt export pump gene (*Bsep*) that is responsible for secreting conjugated bile acids into the bile canaliculi^32,33^ was significantly downregulated in the LKO group. The genes of sodium-dependent taurocholate cotransporting polypeptide (*Ntcp*) and organic anion-transporting polypeptide 1 (*Oatp1*) involved in bile acid uptake were significantly downregulated in the LKO group.

To further confirm the regulation of *Cyp8b1* by GHR, we examined *Cyp8b1* expression level in rhGH treated HFD-fed mice and human HepG2 cell line. *Cyp8b1* expression was significantly reduced in both the rhGH treated mice and HepG2 cells (Figure 5b-c). It is well established that CYP8B1 is regulated by liver FXR, and the expression level of *Fxr* as well as its downstream *Shp* was decreased in the liver of LKO mice (Figure 5d). Therefore, we checked the response of *Cyp8b1* to FXR activation in the LKO mice treated by FXR agonists GW4064. Although *Fxr* and its targeted genes *Shp* and *MafG* were induced by GW4064 (Figure 5e), the expression level of *Cyp8b1* was not changed (Figure 5f). In contrast, although the expression level of *Cyp7a1* was not changed in the rhGH treated mice and HepG2 cells, it was significantly induced in the GW4064 treated LKO mice (Figure S5A-C). Our results suggested that hepatic GH signaling directly regulates *Cyp8b1* independent of FXR.

### Hepatic *Ghr* absence influences gut bile acid profile

**Figure 6.**
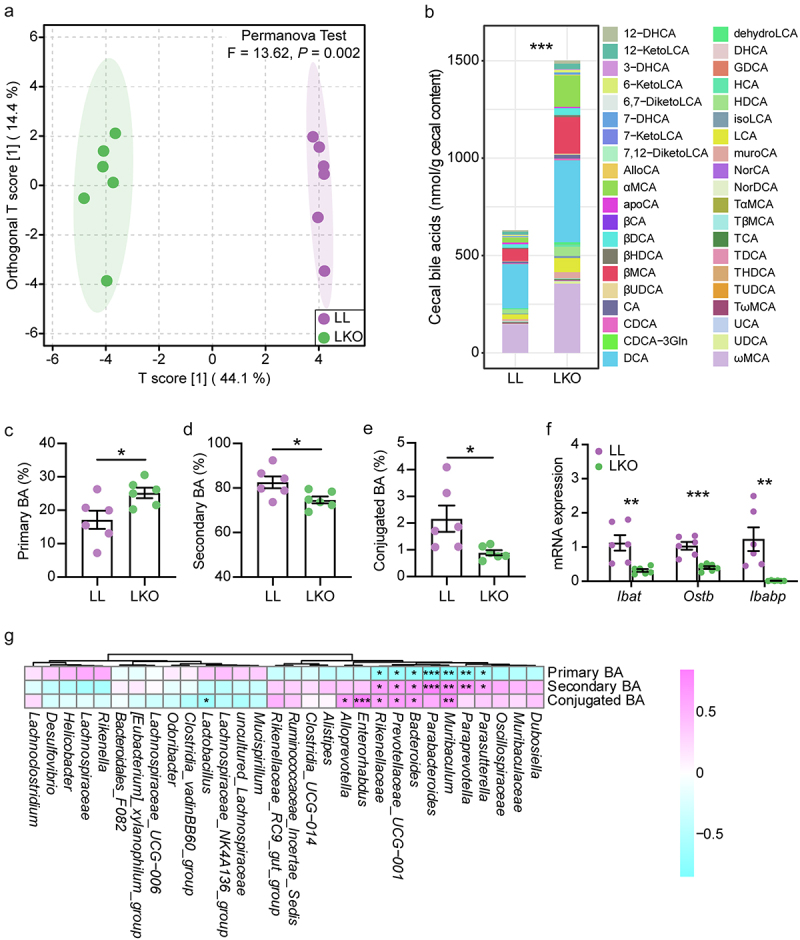
Bile acid metabolism in the cecum and its correlation with gut bacteria. (a) OPLS-DA plot showing the difference of bile acid profile between the LL and LKO mice. (b) Composition of the cecal bile acid pool in the LL and LKO mice. (c-e) Comparison of the abundance of primary bile acid (c), secondary bile acid (d) and conjugated bile acid (e) between the LL and LKO mice. (f) Expression level of the genes related to bile acid reabsorption. (g) Correlation between the bile acid and the top 30 most abundant bacterial genera. *: *P* < 0.05, **: *P* < 0.01, ***: *P* < 0.001.

The bile acid profiles in the cecal content of LL and LKO mice were determined. The OPLS-DA plot displayed a clear discrimination between the two groups, which was verified by the PERMANOVA test with a *P* value of 0.002 (Figure 6a). Both the cecal bile acid pools of the two groups were dominated by the unconjugated bile acids, such as DCA, ωMCA, βMCA, αMCA, and LCA. The contents of these unconjugated bile acids were increased in the LKO group, which contributed to a significant increase of the total bile acid content (Figure 6b, Table S4). In addition, the abundance of the primary bile acid was significantly increased, and those of the secondary bile acid and conjugated bile acid were significantly decreased in the LKO group (Figure 6c-e). We further analyzed the expression level of bile acid transport-related genes in the cecum. It was found that the expression levels of apical sodium dependent BA transporter gene (*Ibat*), organic solute transporter β gene (*Ostb*) and ileal bile acid-binding protein gene (*Ibabp*) were significantly downregulated in the LKO group (Figure 6f). In addition, the expression level of *Fxr* as well as its downstream *Fgf15* was reduced in the cecum of LKO mice (Figure S5D).

The correlation between the abundance of bacterial genus and bile acid was analyzed, and several genera were found to have significant association with the bile acid variation in the cecal content. *Parasutterella*, *Paraprevotella*, *Muribaculum*, *Parabacteroides*, *Bacteroides*, *Paraprevotellaceae UCG-001* and *Rikenellaceae* were significantly negatively correlated with primary bile acid and significantly positively correlated with secondary bile acid. *Muribaculum*, *Bacteroides*, *Paraprevotellaceae UCG-001*, *Rikenellaceae*, *Enterorhabdus*, and *Alloprevotella* had the significantly positive correlation, while *Lactobacillus* had the significantly negative correlation with the conjugated bile acid (Figure 6g).

### The putative function of gut microbiota is changed in hepatic *Ghr* knockout mice

**Figure 7.**
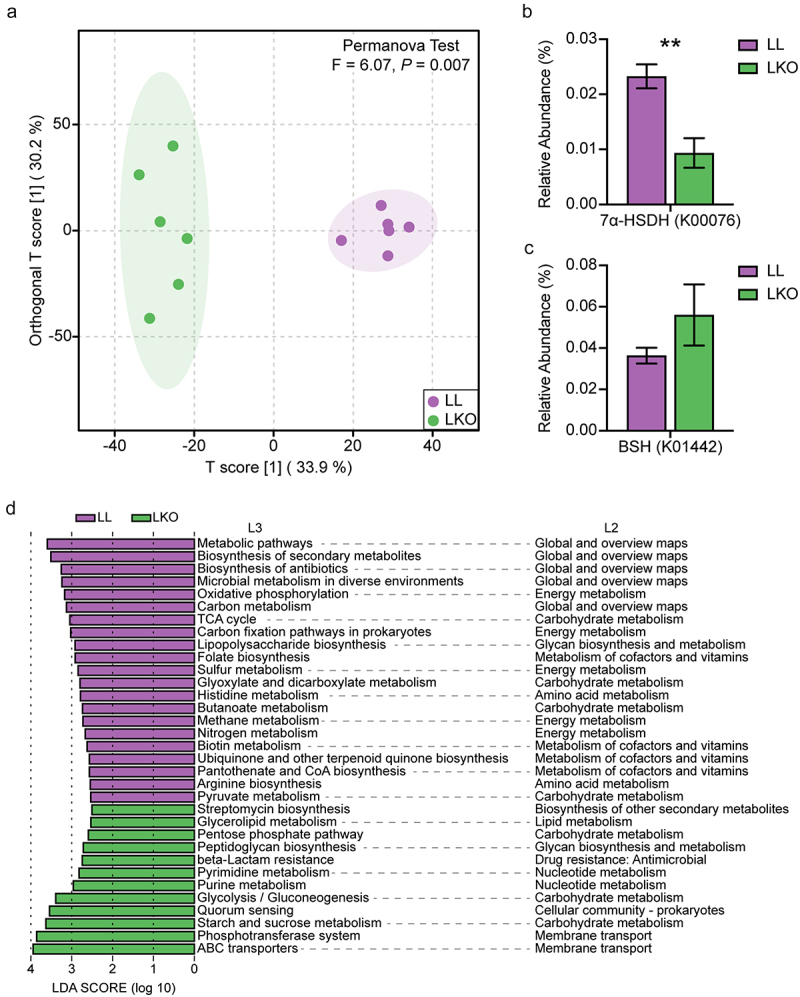
Prediction of gut microbiota function by Tax4Fun2. (a) OPLS-DA plot showing the difference of gut microbiota function at the KO level in the LL and LKO mice. (b-c) Comparison of the relative abundance of 7α-HSDH gene (b) and BSH gene (c) between the LL and LKO mice. *: *P* < 0.05, **: *P* < 0.01, ***: *P* < 0.001. (d) LDA analysis revealing the differentially enriched functional pathways between the LL and LKO mice.

To assess the fluences of hepatic *Ghr* absence on the microbiota function, the function of gut microbiota was predicted by Tax4Fun2 based on the abundance of bacterial ASVs. The OPLS-DA along with PERMANOVA test at the KO level showed that the function of the microbial community was significantly different between the LL and LKO groups (Figure 7a). The abundance of bile acid metabolism-related pathway was analyzed. The abundance of microbial 7α-hydroxysteroid dehydrogenase (7α-HSDH) gene, which takes part in transforming the primary bile acid into the secondary bile acid, was significantly lower in the LKO groups compared with the LL group (Figure 7b). The abundance of microbial bile salt hydrolase (BSH) gene involved in the deconjugation of conjugated bile acid showed a trending increase in the LKO group (Figure 7c). Moreover, 33 significantly altered KEGG L3 pathways were identified between the LL and LKO groups by LDA analysis, and 29 of them affiliates with the “Metabolism” pathway at L1 category (Figure 7d), mainly including “Global and overview maps”, “Energy metabolism”, “Carbohydrate metabolism” pathways in the L2 category. Among them, several pathways associated with short chain fatty acid (SCFA) production were significantly downregulated in the LKO group, including “Glyoxylate and dicarboxylate metabolism”, “Butanoate metabolism”, and “Pyruvate metabolism”, which suggested the altered gut microbiota affected the gut SCFAs.

### Microbial metabolite levels are altered in hepatic *Ghr* knockout mice

**Figure 8.**
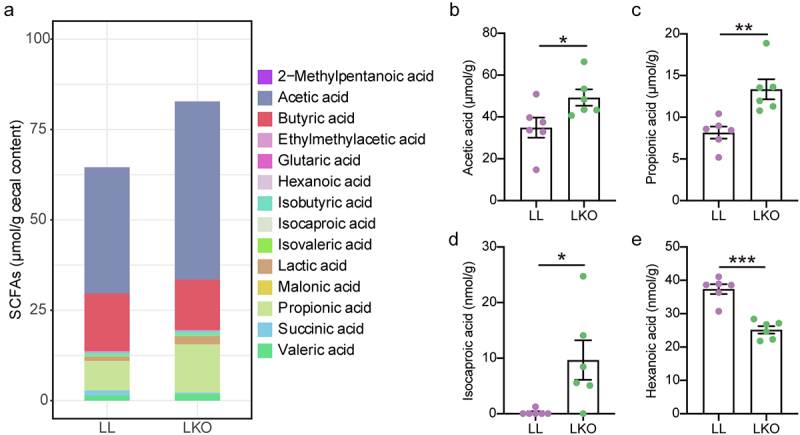
Analysis of the SCFA profile in cecal content of the LL and LKO mice. (a) Composition of SCFA pool in the LL and LKO mice. (b-e) Comparison of the individual SCFA content in the LL and LKO mice. *: *P* < 0.05, **: *P* < 0.01, ***: *P* < 0.001.

The SCFAs in cecal content were detected in the LL and LKO mice to confirm the Tax4Fun2 prediction. A total of 14 SCFAs were identified (Table S5), which was dominated by acetic acid, butyric acid and propionic acid, and there was a trending increase in the content of total SCFA in the LKO group without significance (Figure 8a). Moreover, the levels of four SCFAs were significantly changed. The contents of acetic acid, propionic acid and isocaproic acid were significantly increased, and that of hexanoic acid was significantly decreased in the LKO group (Figure 8b-e).

**Figure 9.**
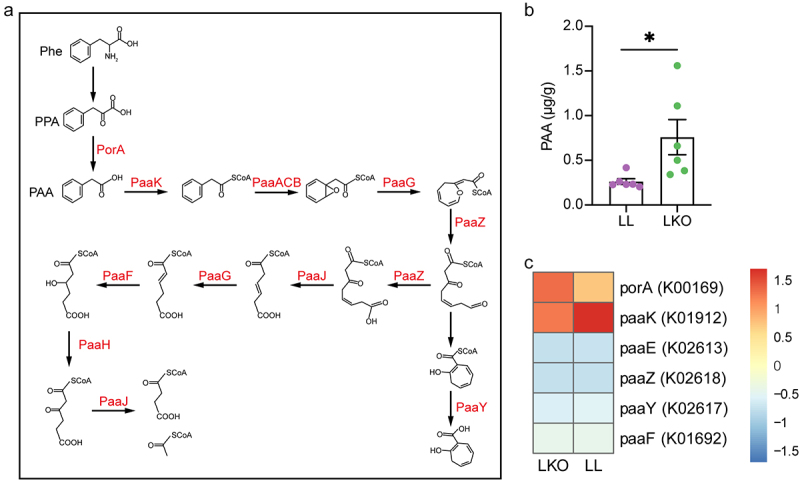
PAA metabolism in the LL and LKO mice. (a) Schema of bacterial PAA metabolism pathway. (b) Comparison of liver PAA content between the LL and LKO mice. (c) Relative abundance of PAA metabolism genes predicted by Tax4Fun2 in the cecal content of LL and LKO mice.

We also observed the alteration of another microbial metabolite – phenylacetic acid (PAA), which is closely related to fatty liver. PAA is produced by the bacterial fermentation of phenylalanine (Phe). It is metabolized from phenylpyruvic acid (PPA) by PorA and degraded by several enzymes or enzymatic subunits, such as PaaK, PaaABC, and PaaG, which are encoded by the genes in the paa operon (Figure 9a). In this study, PAA content was significantly increased in the liver of LKO mouse (Figure 9b). The abundances of genes involved in PAA metabolism in gut microbiota were analyzed based on Tax4Fun2 prediction. *porA* and *paaK* were abundant in both the LL and LKO group, but the abundance of *paaK* was relatively higher than *porA* in the LL group (Figure 9c), suggesting a stronger PAA degradation ability of the gut microbiome in the LL group.

## Discussion

In this study, we characterized the gut bacterial community in mice with GHR deleted in the adipose tissue and liver that are the important targets of GH. Neither of them influenced the richness and evenness of the bacterial community compared with the LL control, which was consistent with the results in GH^−/−^ mice.^19^ Together with our finding, we concluded that the effect of GH action on gut microbiota α-diversity is mild. However, hepatic GHR absence led to significant change in the taxonomic composition of gut microbiota compared with LL and AKO groups. The abundances of *Firmicutes* and *Bacteroidota* dominated in murine gut microbiome as well as many genera belonging to them were significantly changed in the LKO mice. Interestingly, they were not changed in the global GH overexpress or GH gene knockout mice,^19,20^ which suggested the compensatory effect of GH action in different organs. Moreover, *Parasutterella* significantly reduced in the LKO mice showed the same and opposite trends with that in GH^−/−^ and GH transgenic mice,^19^ respectively. *Parasutterella* has been considered as a core member of the murine and human gut microbiota.^34^ It was characterized to alter multiple biological processes and pathways, such as amino acids, SCFA, and bile acid metabolism, and was also implied to exert the protective effects on liver healthy.^35,36^ This was in line with our correlation analysis, which showed that *Parasutterella* together with several other genera was negatively correlated with the triglyceride and cholesterol levels in the LKO mice. Therefore, these features of *Parasutterella* renders it a potential target for the metabolic diseases associated with dysregulated GH signaling. Overall, GHR disruption in the liver rather than adipose tissue resulted in the significant variation of gut microbiota composition, and the variation of gut microbiota was closely associated with the disturbed lipid homeostasis in the LKO mice.

The disturbed bile acid metabolism in the LKO mice contributed to the gut microbiota variation. Hepatic GHR disruption resulted in the induction of bile acid, which was probably owed to the induction of CYP8B1, a crucial regulator determining the ratio of 12-OH bile acids to non-12-OH bile acids. FXR is an important regulator that negatively regulates CYP8B1. Although *Fxr* was downregulated in the LKO mice, the expression level of *Cyp8b1* was not changed after GW4064 treatment, suggesting that the hepatic GH signaling directly regulates *Cyp8b1* independent of FXR. Surprisingly, *Cyp7a1* that is known to be inhibited by activated FXR was significantly induced in the GW4064 treated LKO mice, implying that there may be a crosstalk between GHR and FXR. The total bile acid content was increased in the gut, which probably attributed to the decreased expression of genes involved in bile acid reabsorption in the gut (*Ibat*, *Ostb*, and *Ibabp*) and uptake in the liver (*Oatp1* and *Ntcp*). Bile acids can inhibit the bacterial growth, and some bacteria such as *Lactobacillus* and *Clostridium*, are resistant to bile acid.^22,37^ Therefore, the increased bile acid in the gut of LKO mice probably inhibited the growth of many bacteria, such as *Muribaculum*, *Parabacteroides*, *Parasutterella*, and *Bacteroides*. However, the abundance of *Lactobacillus* was significantly increased in the LKO group owing to its bile acid resistance. In addition, the suppressed cecal FXR was probably also involved in the disturbed microbiota, since it is regarded to regulate genes participating in enteroprotection.^23^ Moreover, the variation of these bacteria in turn resulted in the lower abundance of secondary bile acid and conjugated bile acid in the LKO mice, since *Muribaculum*, *Parabacteroides*, *Parasutterella*, and *Bacteroides* have been found to produce secondary bile acids^35,38,39^ and *Lactobacillus* is the main genera involved in bile acid deconjugation.^24,33^ This was also in line with the correlation analysis and bacterial community function prediction and indicated that the abundance of 7α-HSDH and BSH genes was affected by the hepatic GHR disruption.

The altered gut microbiota may contribute to the disturbed metabolic phenotypes of the LKO mice through the microbial metabolites. Our results together with previous studies^29,40^ have proved that both the congenital and adult-onset liver GHR disruption result in insulin resistance and hepatic steatosis. However, the underlying microbial mechanism was far from clear. In this study, many bacteria were correlated with the fatty liver phenotype, and several microbial metabolites including SCFAs and PAA that can regulate host metabolism were changed in the LKO mouse. Acetic acid and propionic acid were significantly enriched, while hexanoic acid level was significantly decreased in the LKO mice. Acetic acid and propionic acid are the most abundant SCFAs in the gut, and they were found to be enriched in NAFLD patients.^41^ And, propionic acid was also considered to lead to insulin resistance and hyperinsulinemia.^42^ Hexanoic acid has been reported to inhibit the colonization of potentially pathogenic bacteria in the gut,^43^ and it is also negatively related with Crohn’s disease activity.^44,45^ Besides the SCFAs, the level of gut microbiota derived PAA was increased in the liver of LKO mice, which probably resulted from the inhibition of PAA degradation of the gut microbiota. In a human cohort study, PAA was found to be most strongly associated with hepatic steatosis and proved to induce the lipid accumulation in primary human hepatocytes by reducing the response to insulin via lowering AKT phosphorylation.^46^ Our results suggested that the increased bacterial production of SCFAs and PAA may contribute to the insulin resistance and liver lipid accumulation in the LKO mice, and their detailed effects and underlying mechanisms need to be further studied.

In conclusion, our study proposed the role of hepatic GHR in modulating gut microbiota by regulating bile acid metabolism via CYP8B1. And, the altered gut microbiota in turn affects the bacterial metabolites that may contribute to the insulin resistance and liver lipid accumulation caused by liver GHR disruption. Our findings provide the metabolic basis related to gut microbiota dysbiosis driven by liver GH signaling, which extends our insight into functional roles of GH signaling and facilitates the investigation of GH-gut microbiota crosstalk.

## Materials and methods

### Animal studies

Three genotypes of male mice from a pure C57BL/6J background were used in this study. The adipose tissue-specific *Ghr* knockout mice (AKO) were generated by breeding the GHR^flox/flox^ mice^47,48^ to Adipoq-Cre mice as previously described.^28^ The hepatic tissue-specific *Ghr* knockout mice (LKO) were generated by breeding the GHR^flox/flox^ mice to Albumin-Cre mice (B6.FVB(129)-Tg(Alb1-cre)1Dlr/J, Jackson Laboratory, Stock No. 016832). The GHR^flox/flox^ (LL) mice were used as control. All the mice were fed on regular chow (1010009, 12.79% kcal from fat, Xietong Bioengineering Company, Nanjing, China) in IVC system with 12-hr light/12-hr dark cycles and given *ad libitum* access to food and water. Genotyping of the mice were performed 4 weeks after birth from tail snips by PCR using the primers listed in Table S6. The LKO mice were also treated with either vehicle or FXR agonist GW4064 (M2018, AbMole, TX, USA) by intraperitoneal injection once daily at 30 mg/kg body weight for 14 days.

Male wild type C57BL/6J mice were used for recombinant human growth hormone (rhGH) administration assay. Weaning mice of four-weeks old (Strain No. N000013) were purchased from GemPharmatech (Nanjing, China). After 1 week of acclimation, mice were fed a high fat diet (HFD) containing 60% kcal form fat (XTHF60–1, Xietong Bioengineering Company) for 12 weeks. Then, HFD-fed mice were divided into two groups: (1) the GH group treated by daily intraperitoneal injection with 0.75 IU/kg body weight rhGH (AnkeBio Co., Ltd, Anhui, China), and (2) the control group treated by intraperitoneal injection with normal saline. Mice were sacrificed after 4 weeks of rhGH administration assay. All animal experiments were performed under the guidelines for the treatment of laboratory animals and were approved by the Committee on the Ethics of Animal Experiments of Shandong Provincial Hospital Affiliated to Shandong First Medical University & Shandong Academy of Medical Sciences.

### Sample collection and processing

Liver, adipose tissue, blood, cecum, and cecal content were sampled from the 20-week-old mice. The liver, cecum, white adipose tissue (WAT) and brown adipose tissue (BAT) were dissected. After being weighed, partial liver and subcutaneous fat were fixed with 10% formalin for histological observation, and the remaining liver and adipose tissue samples together with the cecum samples were snap-frozen in liquid nitrogen. Serum was separated from whole blood by centrifugation at 1000 g for 10 min at 4°C and stored at −80°C for further use. Cecal contents were collected and snap-frozen immediately and stored at −80°C for gut bacterial community analysis.

### Body weight, organ weight, and biochemical parameters analysis

Body weight of each mouse was recorded before sacrifice. Liver and fat were weighed after dissection using an analytical balance. The content of triglyceride, total cholesterol, low-density lipoprotein cholesterol, and high-density lipoprotein cholesterol in the serum or liver was measured using the test kits (A110-1-1, A111-1-1, A113-1-1 and A112-1-1, Jian Cheng Biological Engineering Institute, Nanjing, China) according to the manufacturer’s instructions.

### Insulin tolerance test

At the end of the experiment, insulin tolerance test (ITT) was performed as described previously^47^. Briefly, blood glucose was first measured after the mice were fasted for 4 h. Subsequently, 0.75 international units (IU) per kg insulin (Humulin R, Eli Lily, Indianapolis, IN) was injected intraperitoneally into mice and blood glucose level was measured at 15-, 30-, 45- and 60-min post insulin injection. The area under the curve (AUC) was calculated as described previously.^49^

### Hematoxylin–Eosin (H&E) staining

Liver and fat tissues were fixed with 10% formalin for at least 24 h. Samples were sliced into 5 μm sections after embedding in paraffin and then stained with H&E. Digital images were captured with a Nikon NI-E light microscope (Nikon, Tokyo, Japan).

### HepG2 cell treatments

Human HepG2 cell line was maintained in Dulbecco’s modified Eagle’s medium (11330032, Gibco, NY, USA) supplemented with 10% fetal bovine serum (GeminiBio, CA, USA). In rhGH treatment assay, cells were treated with rhGH at 160 ng/mL for 24 h, and those treated with normal saline were used as control.

### High-throughput sequencing of 16S rRNA gene

The total DNA in the cecal content was extracted with the E.Z.N.A. stool DNA isolation kit (OMEGA Bio-tek, GA, USA) according to the manufacturer’s instruction. The hypervariable V3–V4 region of 16S rRNA gene was amplified with the universal primers of 343F (5′-TACGGRAGGCAGCAG-3′) and 798 R (5′-AGGGTATCTAATCCT-3′) using the purified DNA as template. The amplicons were then pair-end sequenced on the Illumina MiSeq PE250 platform by Majorbio Bio-pharm Technology Co., Ltd (Shanghai, China).

### Analysis of bacterial community in cecal content

The raw sequencing data were processed in QIIME2^50^ to analyze the diversity and taxonomic composition of the bacterial community. Briefly, the paired-end reads were denoised and stitched by DADA2 after demultiplexing and quality examination, which generated an amplicon sequence variant (ASV) table along with the representative sequences. In the diversity analysis, the ASV table was subsampled based on the minimum count of all the samples to avoid bias caused by different sequencing depths. The α- and β-diversity analyses were performed on the normalized ASV table. The β-diversity was illustrated by 3D plot based on the principal component analysis (PCA), and permutation on a multivariate analysis of variance (PERMANOVA) was conducted to test the statistical significance between different groups. The taxonomical classification was performed to analyze the relative abundance of bacterial species in different groups. The taxonomy was first assigned to the representative sequences with a classifier trained on Silva 138 99% 16S reference, and then the taxonomic composition of bacterial community in each sample was analyzed based on the ASV table. The taxonomic composition was visualized by stack bar charts and heatmap at phylum and genus level plotted by “ggplot2” and “pheatmap” in R software.

### Prediction of the bacterial community function

The functional capabilities of bacterial community were predicted using Tax4Fun2 package^51^ in R software based on the ASVs and the representative sequences obtained by QIIME2. The Kyoto Encyclopedia of Gene and Genomes (KEGG) database was used as the reference, and the KEGG Orthologs (KOs) were predicted based on the relative abundance of all the ASVs. The KEGG pathway abundance was consequently inferred based on the KOs abundance. Orthogonal partial least-squares discrimination analysis (OPLS-DA) and PERMANOVA were performed to analyze the variation of the functional profile of the bacterial community. Linear discriminant analysis (LDA)^52^ was conducted to identify the significantly altered functional pathways.

### Targeted quantitative metabolomics, bile acid, and short chain fatty acid (SCFA) profile analyses

Frozen samples were sent to Metabo-Profile (Shanghai, China) for targeted metabolomics analysis. The adipose tissue and liver samples of LL, AKO, and LKO groups were evaluated by targeted quantitative metabolomics on the Q300^TM^ primary targeted metabolic profiling platform using triple quadrupole mass spectrometer with an ultrahigh performance liquid chromatography (UPLC-QQQ-MS), which focuses on ~300 targeted metabolites such as amino acids, fatty acids, bile acids, organic acids, carbohydrates, lipids, etc. The bile acid profiles in the serum and cecal content of LL and LKO mice were determined by the ultrahigh performance liquid chromatography-tandem mass spectrometry (UPLC-MS/MS) using the BAP Ultra kit (Metabo-Profile, Shanghai, China). The detection of cecal SCFAs was also performed by Metabo-Profile using UPLC-MS/MS. OPLS-DA and principal component analysis (PCA) were performed to analyze the differences of metabolite profile between different groups, and PERMANOVA was used to determine the statistical significance.

### RNA extraction and gene expression assessment

Total RNA was extracted from liver and cecum using TRIzol reagent (9109; Takara, Beijing, China), and then reverse-transcribed into cDNA using First Strand cDNA Synthesis Kit (SM134, Sevenbio, Beijing, China). The relative expression levels of genes involved in bile acid metabolism and transport were quantified by SYBR Green qPCR MasterMix (SM143, Sevenbio) with *Gapdh* gene as the reference on the QuantStudio 7 Pro Real-Time PCR System (Applied Biosystems, CA, USA). The primer sequences are listed in Table S6.

### Statistical analysis

All values are shown as mean ± SEM. T-test between groups and one-way ANOVA analysis followed by Tukey’s post hoc test among LL, LKO, and AKO groups were performed. The statistical significance threshold was set at *P* < 0.05. The statistical significance was indicated by * (*P* < 0.05), ** (*P* < 0.01), and *** (*P* < 0.001).

The pairwise Pearson’s correlations between the abundances of bacterial genera and metabolic phenotype and bile acids were calculated by “psych” and visualized by “pheatmap” in R software.

Weighted gene co-expression network analysis (WGCNA)^53^ was performed to identify the key gut bacteria-related metabolic modules and metabolites using the WGCNA package in R software. Firstly, the metabolites from adipose tissue and liver were, respectively, clustered using hierarchical clustering and topology overlap measures (TOM) based on co-expression correlation coefficient of each metabolite. Then, module-trait association analysis was conducted to calculate the Pearson correlation coefficient between metabolic modules and bacterial phyla, and the most relevant and significant metabolic module was selected and detailed. Next, the module membership (MM) and the biomarker significance (BS) were calculated for each node in each module.

## Supplementary Material

Supplemental Material

## Data Availability

The raw sequence data are available in the NCBI Sequence Read Archive database under accession number PRJNA913256 (http://www.ncbi.nlm.nih.gov/sra). The targeted metabolomics data are included in the supplementary files.
