# Supplementary material for: Deletion of hepatic growth hormone receptor (GHR) alters the mouse gut microbiota by affecting bile acid metabolism
Source: Gut Microbes. 2023 Jun 12;15(1):2221098. doi: 10.1080/19490976.2023.2221098 (PMC10262758; doi:10.1080/19490976.2023.2221098)
Supplement: Supplemental Material [file KGMI_A_2221098_SM7730.zip › Supplemental material_KMAB_2221098/Supplementary Figure S1.docx]

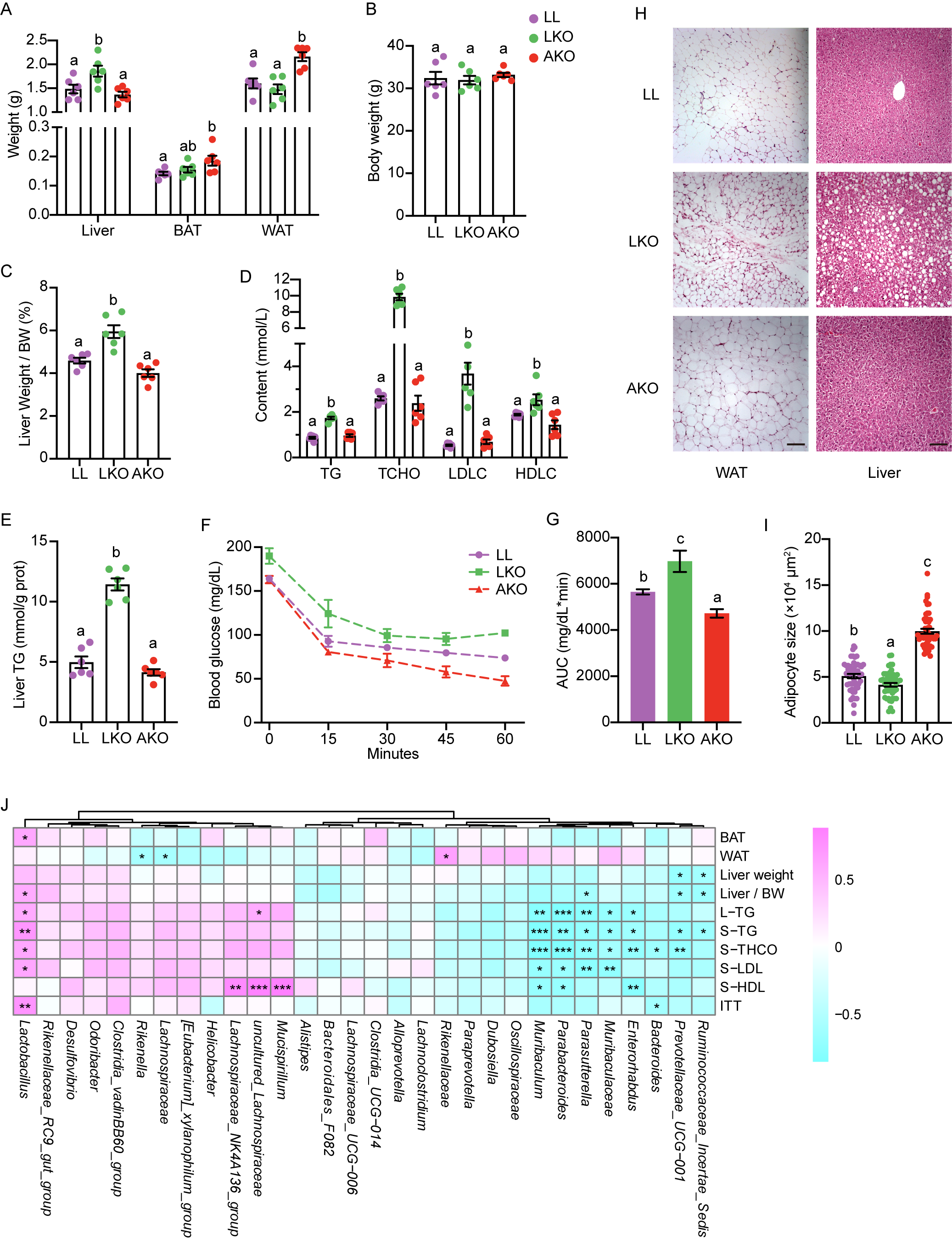


**Figure S1** Metabolic phenotype of LL, LKO and AKO mice and its correlation with gut microbiota. A: Weights of the liver, brown adipose tissue (BAT) and white adipose tissue (WAT). B: Body weight of the LL, LKO, and AKO mice. C: Liver weight / body weight (BW) ratio. D: Content of triglyceride (TG), total cholesterol (TCHO), low-density lipoprotein cholesterol (LDLC) and high-density lipoprotein cholesterol (HDLC) in the serum. E: TG content in the liver. F: Blood glucose levels of ITT. G: AUC of ITT. H: H&E staining of the subcutaneous WAT and liver. Bar: 100 μm. I: adipocyte size analysis. J: Correlation between the metabolic indices and gut bacteria in the LKO mice. L-: Liver, S-: Serum. Different lowercase letters above the error bars indicate significant differences among groups. *: *P* < 0.05, **: *P* < 0.01, ***: *P* < 0.001.
