# Supplementary material for: Deletion of hepatic growth hormone receptor (GHR) alters the mouse gut microbiota by affecting bile acid metabolism
Source: Gut Microbes. 2023 Jun 12;15(1):2221098. doi: 10.1080/19490976.2023.2221098 (PMC10262758; doi:10.1080/19490976.2023.2221098)
Supplement: Supplemental Material [file KGMI_A_2221098_SM7730.zip › Supplemental material_KMAB_2221098/Supplementary Figure S2.docx]

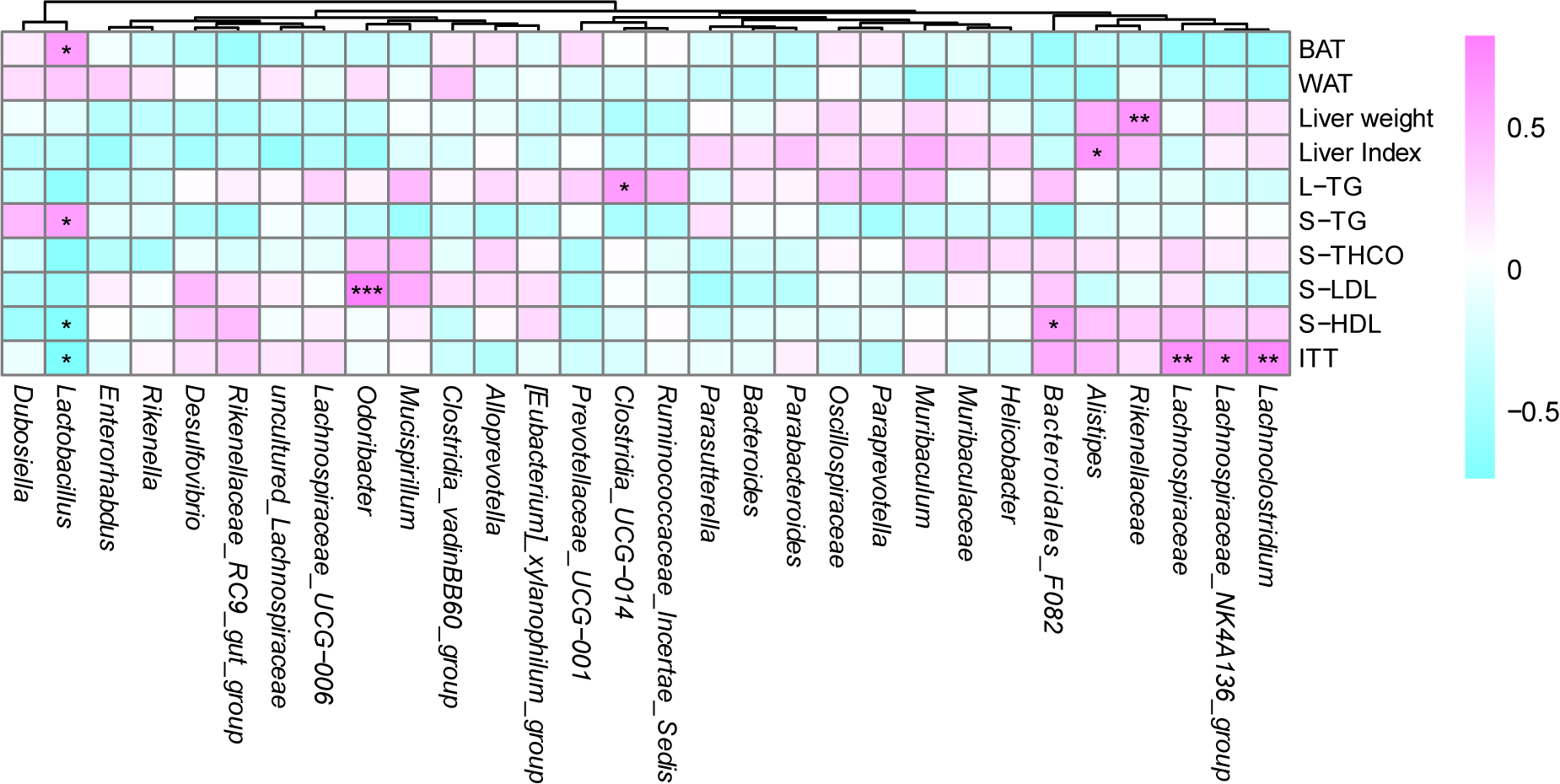


**Figure S2** Correlation between the metabolic indices and gut bacteria in the AKO mice. L-: Liver, S-: Serum. *: *P* < 0.05, **: *P* < 0.01, ***: *P* < 0.001.
