# Supplementary material for: Deletion of hepatic growth hormone receptor (GHR) alters the mouse gut microbiota by affecting bile acid metabolism
Source: Gut Microbes. 2023 Jun 12;15(1):2221098. doi: 10.1080/19490976.2023.2221098 (PMC10262758; doi:10.1080/19490976.2023.2221098)
Supplement: Supplemental Material [file KGMI_A_2221098_SM7730.zip › Supplemental material_KMAB_2221098/Supplementary Figure S3.docx]

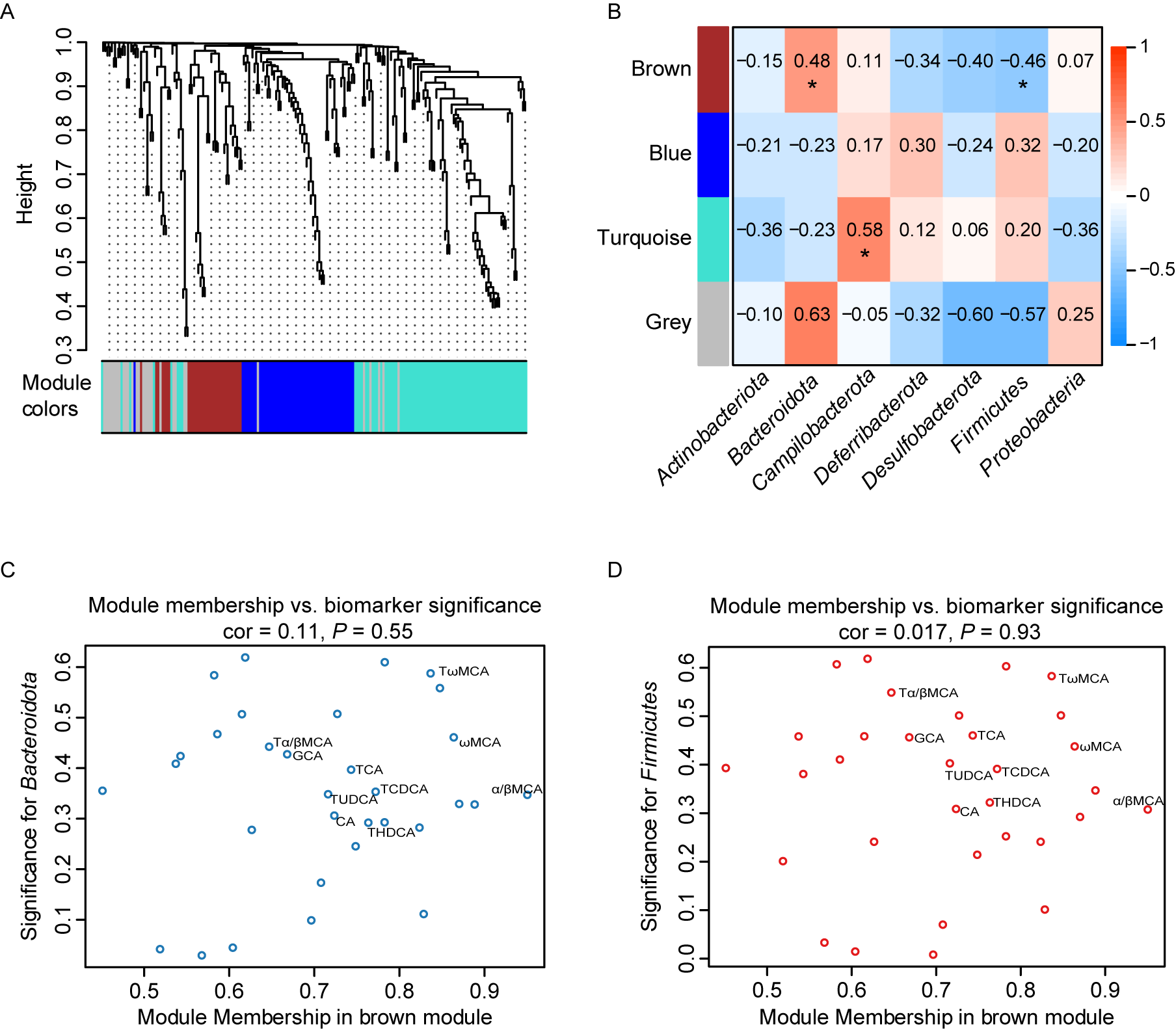


**Figure S3** Correlation analysis of gut bacterial phyla and metabolites in adipose tissue by WGCNA. A: Metabolite modules obtained based on the 196 metabolites in the adipose tissue of LL, LKO, and AKO mice. B: Heatmap presenting module-trait relationships based on the Pearson method. ﻿Each row corresponds to a module eigengene (ME) and each column to the abundance of a bacterial phylum. ﻿The corresponding correlation coefficient is displayed at the top of the cell, and corresponding p-value for each module is displayed by the star. *: *P* < 0.05, **: *P* < 0.01, ***: *P* < 0.001. C-D: The relationship between module membership in the blue module and biomarker significance of *Bacteroidota* (C) and *Firmicutes* (D).
