# Supplementary material for: Deletion of hepatic growth hormone receptor (GHR) alters the mouse gut microbiota by affecting bile acid metabolism
Source: Gut Microbes. 2023 Jun 12;15(1):2221098. doi: 10.1080/19490976.2023.2221098 (PMC10262758; doi:10.1080/19490976.2023.2221098)
Supplement: Supplemental Material [file KGMI_A_2221098_SM7730.zip › Supplemental material_KMAB_2221098/Supplementary Figure S4.docx]

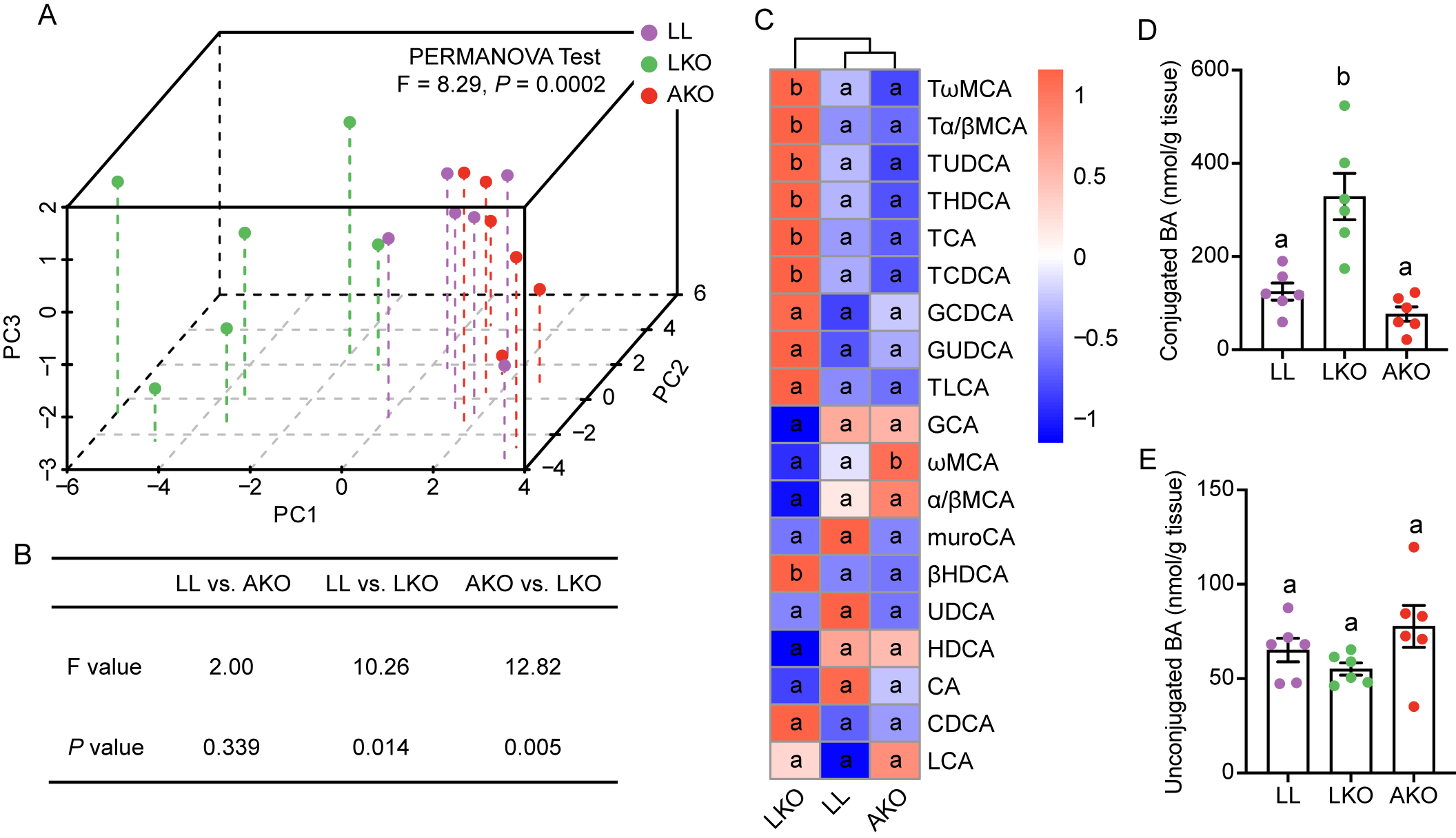


**Figure S4** Change of the live bile acid profile in LL, LKO and AKO mice. A: PCA analysis of the bile acid profile. B: PERMANOVA test of the bile acid profile between different groups. C: Heatmap displaying the distribution of each bile acid in different mice. D: Comparison of conjugated bile acid content in different mice. E: Comparison of unconjugated bile acid content in different mice. The lowercase letters in C-E indicate significant difference among groups.
