# Supplementary material for: Deletion of hepatic growth hormone receptor (GHR) alters the mouse gut microbiota by affecting bile acid metabolism
Source: Gut Microbes. 2023 Jun 12;15(1):2221098. doi: 10.1080/19490976.2023.2221098 (PMC10262758; doi:10.1080/19490976.2023.2221098)
Supplement: Supplemental Material [file KGMI_A_2221098_SM7730.zip › Supplemental material_KMAB_2221098/Supplementary Figure S5.docx]

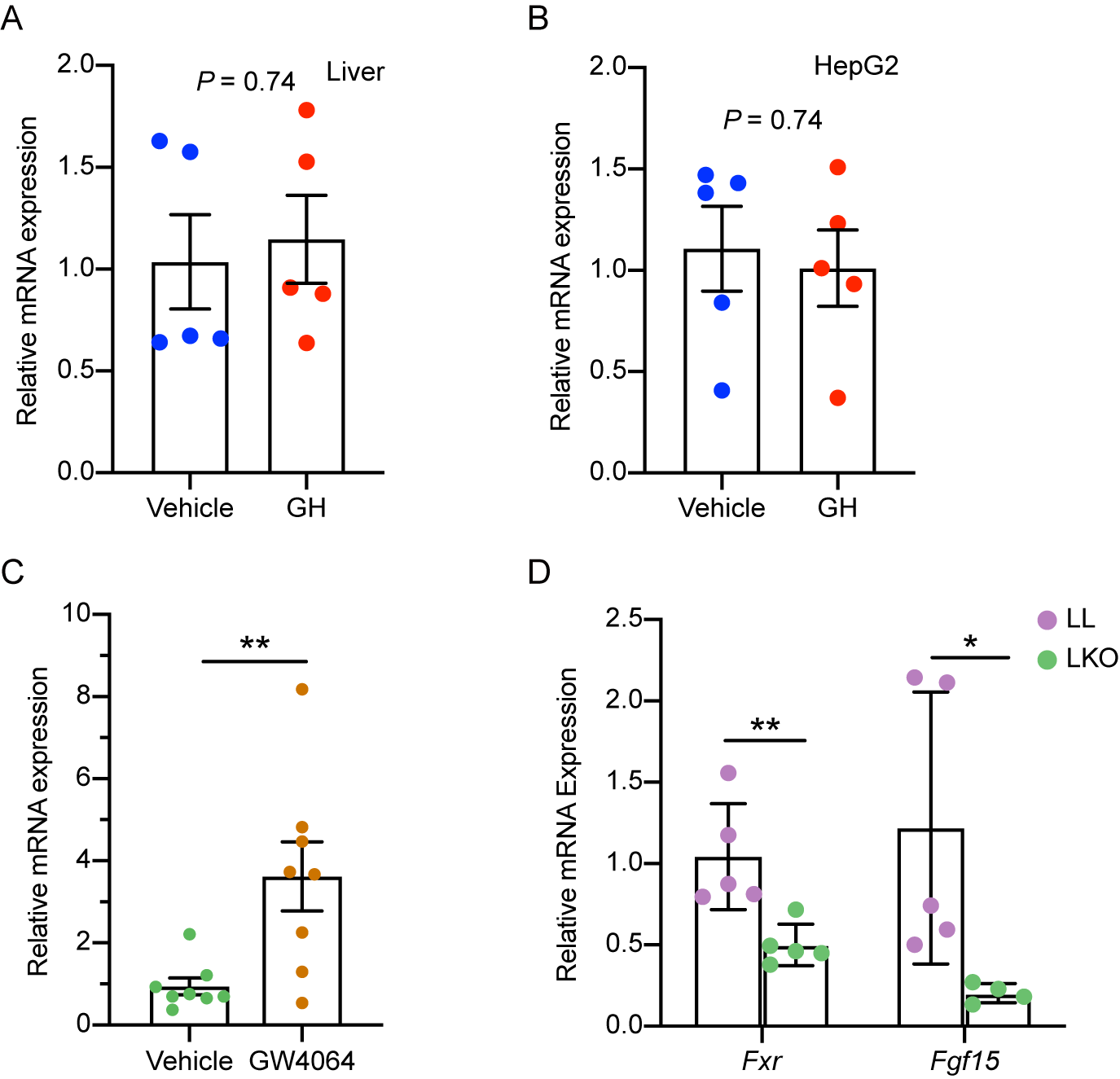


**Figure S5** The expression of genes encoding CYP7A1 in mouse liver and HepG2 cells (A-C) and expression of *Fxr* and *Fgf15* in the cecum of LKO mice. A: Expression level of *Cyp7a1* in rhGH treated HFD fed mice. B: Expression level of *CYP7A1* in rhGH treated HepG2 cells. C: Expression level of *Cyp7a1* in GW4064 treated LKO mice. D: Expression level of *Fxr* and *Fgf15* in the cecum of LKO mice. *: *P* < 0.05, **: *P* < 0.01, ***: *P* < 0.001
